# Single-Cell Technologies Applied to HIV-1 Research: Reaching Maturity

**DOI:** 10.3389/fmicb.2020.00297

**Published:** 2020-03-04

**Authors:** Gérémy Sannier, Mathieu Dubé, Daniel E. Kaufmann

**Affiliations:** ^1^Research Centre of the Centre Hospitalier de l’Université de Montréal (CRCHUM), Montreal, QC, Canada; ^2^Department of Microbiology, Infectiology and Immunology, Université de Montréal, Montreal, QC, Canada; ^3^Department of Medicine, Université de Montréal, Montreal, QC, Canada; ^4^Consortium for HIV/AIDS Vaccine Development (Scripps CHAVD), La Jolla, CA, United States

**Keywords:** HIV-1, single-cell technologies, pathogenesis, cure, vaccine, single-cell omics, fluorescence *in situ* DNA and RNA hybridization, mass cytometry (CyTOF)

## Abstract

The need for definitive answers probably explains our natural tendency to seek simplicity. The reductionist “bulk” approach, in which a mean behavior is attributed to a heterogeneous cell population, fulfills this need by considerably helping the conceptualization of complex biological processes. However, the limits of this methodology are becoming increasingly clear as models seek to explain biological events occurring *in vivo*, where heterogeneity is the rule. Research in the HIV-1 field is no exception: the challenges encountered in the development of preventive and curative anti-HIV-1 strategies may well originate in part from inadequate assumptions built on bulk technologies, highlighting the need for new perspectives. The emergence of diverse single-cell technologies set the stage for potential breakthrough discoveries, as heterogeneous processes can now be investigated with an unprecedented depth in topics as diverse as HIV-1 tropism, dynamics of the replication cycle, latency, viral reservoirs and immune control. In this review, we summarize recent advances in the HIV-1 field made possible by single-cell technologies, and contextualize their importance.

## Introduction

HIV-1 remains a major public health problem around the world. Although ART succeeds in suppressing viral replication and has had a tremendously positive impact for people living with HIV-1, it fails to eradicate the virus and restore effective anti-HIV-1 immunity: the virus persists in long-lived reservoirs, and viral rebound occurs almost invariably after cessation of therapy. HIV-1 pathogenesis is complex and diverse at multiple levels. In the absence of therapy, steady-state viremia and disease progression rate are highly variable, depending on both host and pathogen factors; the enormous diversity of circulating viral strains is a major hurdle for the development of effective vaccination and cure strategies (Ho et al., 2013). This intricacy is also important with regard to immunovirological features within an HIV-1-infected individual. HIV-1 infects or interacts with a wide variety of immune cells that harbor considerable heterogeneity in term of phenotype and functions (Chomont et al., 2009).

Fast evolution, diversification and coordination are core traits allowing immune cells to keep up with the threat of remarkably diverse pathogens. Elucidating this complex interconnected cellular network is a formidable task only achievable through high dimensional tools. Despite the increasing availability of these approaches, single-cell studies on HIV-1 infection remains few relative to other immunology fields. Studying HIV-1-infected cells at the single-cell level remains particularly challenging for various reasons: (1) The extremely low frequency of HIV-1^+^ CD4^+^ T cells, in particular in ART-suppressed individuals (Baxter et al., 2016); the large cell number needed to overcome rare event sampling errors (predicted by the Poisson distribution) and the assay specificity required are often beyond the capacity of many single-cell methods. (2) A large fraction of the integrated HIV-1 DNA proviruses are latent (Ho et al., 2013); currently, no known viral protein or unambiguous cellular surface marker allows their detection in quiescent cells. (3) Secondary lymphoid tissues, which are the main sites of HIV-1 replication and persistence and therefore key for pathogenesis and cure studies, are difficult to sample in humans, thus limiting downstream analyses (Estes et al., 2017). (4) Biosafety issues can make some studies difficult to achieve. Fixation can affect yield and resolution in certain single-cell systems and cutting-edge equipment is not always available in containment labs to work on unfixed samples.

Despite these hurdles, great strides were nonetheless made using more standard methods that could be considered conceptual predecessors of newer single-cell technologies, including limiting dilutions, subpopulation partitioning by population cell sorting, digital droplet PCR (ddPCR), immunohistochemistry, conventional confocal microscopy and flow cytometry etc. While these technologies remain major research tools, their low dimensionality, poor resolution, laboriousness or low-throughput are all good reasons to complement them with newer single-cell techniques. Single-cell “multiomic” technologies play a dominant role in the “single-cell revolution,” but other cutting-edge approaches must not be overlooked. In this review, we broadly define “single-cell technologies” as any approach providing quantitative analyses reaching single-cell resolution. For convenience, we grouped these technologies in four global categories based on their key contribution to the field (Table 1).

**TABLE 1:** Some examples of studies providing single-cell insight into HIV-1 biology or pathogenesis.

	**Approaches**	**Description**	**Few examples of application**
Detection of rare events	Branched DNA signal amplification (RNA or DNA)	Flow cytometric or microscopic detection of RNAs or DNAs, compatible with protein co-detection	Compare latency reversal in different cell subsets (Baxter et al., 2016; Grau-Exposito et al., 2019)
			Quantify and phenotype the viral reservoirs *ex vivo* (Baxter et al., 2016; Grau-Exposito et al., 2017)
			Interrogate viral reservoirs in tissues (Deleage et al., 2016) and estimate whole body viral burden (Estes et al., 2017)
			Identify HIV^+^ cells in tissue-resident cells, including non-T cells (Vasquez et al., 2018)
	Dual protein detection	Co-detection of viral proteins by flow cytometry	Study translation-competent viral reservoirs (DeMaster et al., 2015; Pardons et al., 2019)

Genetic profiling	Targeted PCR for viral genes	Quantification of RNA or DNA targets	Correlate residual HIV-1 transcription to sites of integrated proviruses (Wiegand et al., 2017)
			Quantify HIV-1 splicing upon latency reversal (Yucha et al., 2017)
			Assess gene expression in different stages of SIV replication (Bolton et al., 2017)
	Unsupervised sequencing (RNAseq, DNAseq, and ATAC-seq)	Unbiased assessment of transcriptional and epigenetic landscapes	Identify biomarkers of HIV-1 permissiveness (Rato et al., 2017)
			Define quiescent HIV-1 infected cells (Bradley et al., 2018; Golumbeanu et al., 2018), B cell profile post-vaccination (de Armas et al., 2019)
			Establish an epigenetic signature of resident memory T cells during HIV infection (Buggert et al., 2018)
	BCR and TCR sequencing	Profiling of the B cell and T cell repertoires	Analysis of BCR repertoire post-immunization (Scheid et al., 2009; Sundling et al., 2014)
			Study T cell clonal expansion *in vivo* in the context of HIV infection (Wendel et al., 2018)
	Integration sequencing	Mapping of integrated vDNA	Map HIV-1 integration sites in the CD4^+^ T cell genome of primary samples (Cohn et al., 2015)
	Virus barcoding	Engineered viruses with degenerate unique barcodes	Examine the transcriptional potential of integrations sites by correlating barcodes in integrated DNA and vRNA (Chen et al., 2017)

High dimensional phenotyping	Mass cytometry (CyTOF)	Time-of-flight cytometry based on heavy ion metal tags with minimal spectral overlap	Evaluate the susceptibility of CD4^+^ T subsets to productive HIV-1 infection (Cavrois et al., 2017)
			Define the phenotypic landscape of exhausted T cells (Bengsch et al., 2018a; Bekele et al., 2019)
			Link new CD8^+^ T cell subsets to HIV-1 pathogenesis (Buggert et al., 2018)

Imaging of subcellular molecular dynamics	Fluorescent tags	Temporal interrogation of bioengineered fluorescently tagged proteins of interest in primary cells	Dissect, in live cells viral entry (Miyauchi et al., 2009), uncoating (Arhel et al., 2006; Mamede et al., 2017; Francis and Melikyan, 2018b), nuclear import (Chin et al., 2015), and assembly (Ivanchenko et al., 2009) Estimate the timeline of gene expression (Holmes et al., 2015)
	Branched DNA signal amplification for RNA/DNA single-cell microscopy	Snapshots of selected RNAs, vDNA and proteins sub-localization	Study the nuclear import of vDNA (Chin et al., 2015)
			Locate integration sites of native proviruses in primary cells (Marini et al., 2015)
			Study the uncoating of native viruses (Puray-Chavez et al., 2017)
	Imaging of integrated DNA	SCIP	Investigate the spatial localization of HIV-1 integration sites in live cells (Di Primio et al., 2013)
		Detection of CRISPR-Cas9-cleaved integrated provirus	Assess HIV-1 integration in real-time in live cells (Ma et al., 2017)

### Single-Cell Detection of Rare Events

**FIGURE 1:**
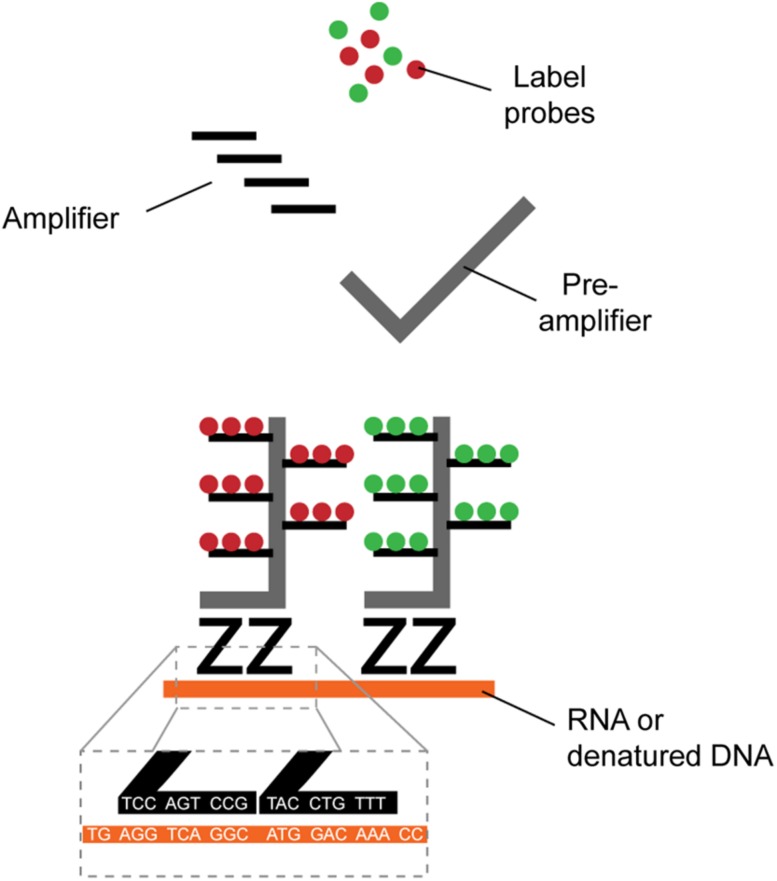
Schematic representation of branched DNA signal amplification technologies. A pair of “Z” probes co-anneal on two 20-mer target RNA or DNA sequences (roots). The flanking regions of the Z probes are next further targeted by a subsequent probe (trunk) on which multiple sites for further fluorescent amplification are present (branches). The extreme improbability of stochastic yet close proximity annealing of two totally independent Z probes and the robust amplification provides excellent signal-to-noise ratio, allowing single-cell detection.

Identification of HIV-1^+^ cells is typically achieved by the detection of viral RNA (vRNA), viral DNA (vDNA) or expression of the structural protein p24. Several direct single-cell virus detection imaging methods with signal amplification were developed in the past years, including *in situ* PCR (Bagasra et al., 1993), tyramide amplification (Soontornniyomkij et al., 1999), and the tunable rolling circle amplification (Frei et al., 2016; Duckworth et al., 2019). All these methods relied on sensitive RNA or DNA fluorescence detection through signal amplification, but at the cost of low reproducibility and high false detection rate due to high background. New methods with higher signal-to-noise ratio combined with dual parametric detection strategies now allow stringent and reliable detection at single-cell resolution (Table 1). The nature of the viral parameters selected for detection impacts data interpretation. CD4 downregulation indirectly provides information about Nef or Vpu expression in the fiber-optic array scanning technology (FAST) assay (DeMaster et al., 2015). Dual non-competitive anti-p24 antibodies (HIVflow) is a convenient way to get insight on p24 translation. The multiplexable branching technology provided the opportunity to use vRNA or vDNA as a co-parameter of detection in fluorescence *in situ* hybridization (FISH) techniques, including RNAflow-FISH (flow cytometry) and DNA/RNAscope (microscopy) assays (Baxter et al., 2016, 2017a,b; Deleage et al., 2016; Estes et al., 2017). This technology takes advantage of the high signal-to-noise ratios of branching RNA or DNA (Figure 1) to achieve high specificity and sensitivity, rapidity and easiness in the structural analysis of HIV-1 reservoirs.

### Single-Cell Genetic Profiling

**FIGURE 2:**
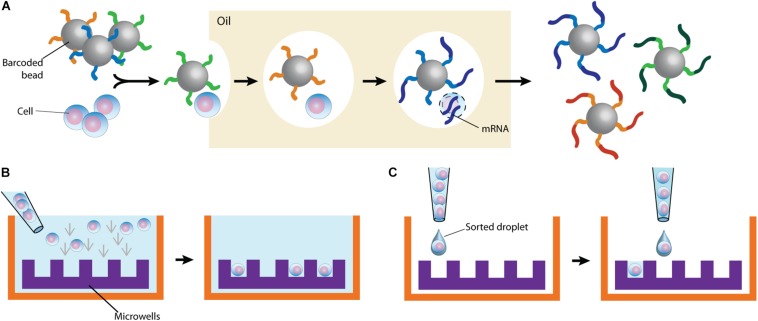
Typical cell partitioning approaches. **(A)** Individual cells and barcoded beads are separated by droplet encapsulation in oil using microfluidic devices. Following intra-droplet cellular lysis, cellular mRNAs are captured by the beads for downstream application. **(B)** Cells are allowed to sediment in wells. To ensure single-cell resolution, sedimentation either occurs at a dilution minimizing doublets or using microwells calibrated to allow deposition of only one cell. **(C)** Single cells are directly sorted in wells. The staining of surface markers provides the mean to enrich for the desired subset of cells.

**FIGURE 3:**
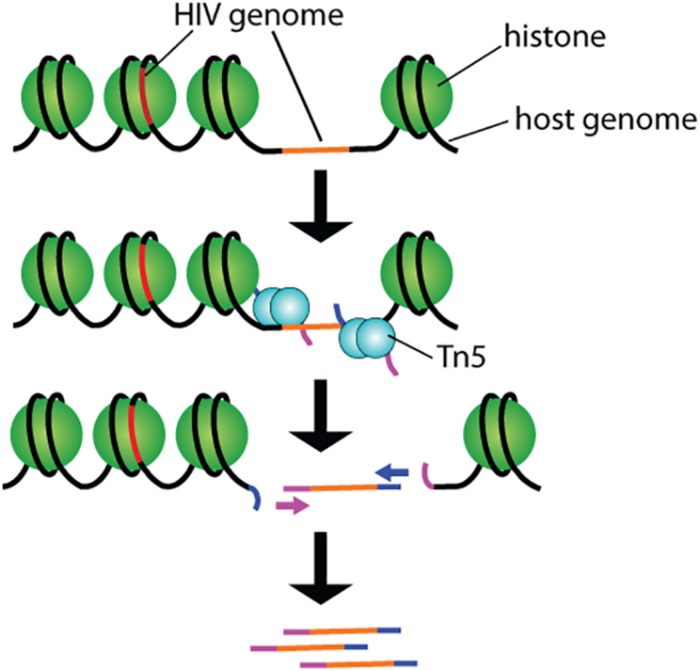
Schematic representation of the ATAC-seq technology. Tn5 transposases preloaded with DNA adapters fragment and tag accessible genomic DNA. Resulting fragments are sequenced and correlated with open and closed chromatin for epigenomic profiling.

By partitioning single cells, capturing their transcripts, and generating sequencing libraries in which the transcripts are mapped to individual cells, single-cell RNA sequencing (scRNA-Seq) and its DNA equivalent (scDNA-Seq) represent unequaled “omic” opportunities. All single-cell sequencing technologies follow the same basic principles. Cells must first be individualized by fluidic technologies, limiting dilutions or single-cell sorting flow cytometry (Figure 2). Single cells are then lysed, and RNA or DNA molecules are amplified to generate a library for deep full-genome sequencing (Rato et al., 2017; Bradley et al., 2018; Golumbeanu et al., 2018; de Armas et al., 2019). Epigenetic profiling at the single-cell level is also possible. The assay for transposase-accessible chromatin using sequencing (ATAC-Seq) enables single-cell epigenomic profiling by taking advantage of the insertion of sequencing adapters by a hyperactive Tn5 transposase mutant to map transcriptionally active chromatin regions (Figure 3; Buggert et al., 2018). Subsequent deep sequencing reveals the degree of transcriptional activity throughout the genome. These methods are increasingly used to study HIV-1 at the single-cell level (Table 1).

### Single-Cell High Dimensional Phenotyping

High-throughput cell phenotyping for protein markers is most frequently performed by polychromatic flow cytometry or mass cytometry (or cytometry by time-of-flight, CyTOF). In addition to antibodies, polychromatic flow cytometry allows detection of fluorescent dyes and benefits from a large pool of commercially available reagents. However, overlapping fluorescence spectra are a recurrent problem that requires complex compensation. Conversely, mass spectrometry by time-of-flight relies on metal-conjugated antibodies requiring essentially no compensation. Limitations of this technology includes fewer available reagents and a lower acquisition throughput than fluorescent cytometry. Current high-end platforms are designed to achieve high dimensionality (up to >30 parameters for cytometers and >40 parameters for CyTOF, accordingly to manufacturers). While technical considerations usually slightly reduce the number of channels useable simultaneously compared to the limit of parameters available, the depth of single-cell profiling achieved is still remarkable (Cavrois et al., 2017; Bengsch et al., 2018a; Buggert et al., 2018; Bekele et al., 2019). For both technologies analytical tools, rather than instrument performance, can still be bottlenecks preventing full exploitation of the data.

### Single-Cell Imaging of Subcellular Molecular Dynamics

Microscopy is often overlooked as a single-cell technology probably because of its traditionally low throughput and semi-quantitative nature. High-resolution time-lapse imaging technologies now provide unprecedented spatial information of unique HIV-1 infected cells in near real-time (Table 1). Automated acquisition and quantification platforms allow unbiased data acquisition, correcting the typical caveat of microscopy. The preservation of the 3D architecture grants access to information impossible to obtain by other single-cell methods.

These recent developments helped the HIV-1 field take the leap toward single-cell technologies. Here, we discuss the contribution of these various new single-cell technologies in the context of HIV-1 research and review concrete examples of their applications.

## HIV-1 Tropism and Host Cell Remodeling

Better defining the nature of HIV-1 infected cells has been an active topic of research since the discovery of the virus. CD4^+^ T cells were quickly found to be primary targets during productive infection. The evolution of molecular biology and flow cytometry tools came with more precise characterization of cells targeted by HIV-1. The ever-improving capacity to divide cell populations into more and more refined subsets by cell sorting or column enrichment enabled the *in vitro* interrogation of various immune cells for either their susceptibility to HIV-1 infection or the presence of vDNA by ultrasensitive PCR methods. These approaches provided a wealth of data, with at times conflicting results that may be mostly due to technical considerations. Numerous studies identified several cell populations with high susceptibility to infection, including central memory CD4^+^ T cells (T_CM_) (Chomont et al., 2009; Jaafoura et al., 2014; Boritz et al., 2016), CD4^+^ T memory stem (T_SCM_) cells (Buzon et al., 2014), regulatory T (T_REG_) cells (Oswald-Richter et al., 2004; Tran et al., 2008; Moreno-Fernandez et al., 2009; McGary et al., 2017); T_H_17 cells (Gosselin et al., 2010, 2017), T follicular helper cells (T_FH_) (Perreau et al., 2013; Banga et al., 2016). All these subsets are defined by sets of phenotypic features broadly accepted at the time of experimentation. However, these labels are not set in stone and are frequently updated based on more refined characterizations. High dimensional single-cell technologies can address these categorizing issues by system biology rather than knowledge-based approaches. For example, unsupervised scRNA-Seq experiments on primary CD4^+^ T cells infected *in vitro* identified novel biomarkers of HIV-1 permissiveness such as CD25, CD298 (ATP1B3), CD63, and CD317 (BST-2) that all correlated with T cell activation (Rato et al., 2017). Activation-induced proteins were not equally predictive of HIV-1 permissiveness, however, suggesting that there are more to permissibility than just cell activation.

Studies of viral permissibility performed *in vitro* must be interpreted with caution. Cultured CD4^+^ T cells drift from their original transcriptional program, especially when exogenous biologically active molecules are applied to maintain survival (ex: IL-2) or promote infection (ex: CD4^+^ T cell activation by PHA or CD3/CD28 crosslinking). Such models are often a reasonable and necessary compromise because of the rarity of infected cells in people living with HIV-1. This bias can now be avoided to some extent by direct *ex vivo* detection of infected cells using dual HIV-1 detection by flow cytometry, as described above. Both HIVflow and RNAflow-FISH showed remarkable consistency in providing *ex vivo* validation of previous bulk observations such as HIV-1 enrichment in cells (1) expressing the activation-associated proteins CD25, HLA-DR, Ki67 and the inhibitory receptors TIGIT, PD-1 or CTLA-4 (Baxter et al., 2016; Pardons et al., 2019); (2) transitional memory CD4^+^ T (T_TM_) cells rather than in central and effector memory (T_CM_ and T_EM_, respectively); (3) in cells of T_H_17, T_FH_ and T_REG_ polarizations (Pardons et al., 2019). Unfortunately, detection of HIV-1 infected cells implies experimental procedures that tend not to preserve well RNA integrity, often precluding downstream RNA-Seq analysis. However, high dimensional protein profiling of infected cells is now doable by flow cytometry and while some obstacles remain, these strategies are adaptable to mass cytometry. These single-cell technologies enable studying the permissibility of subsets to sub-viral processes like entry and gene expression using engineered reporter viruses. In a recent mass cytometry study (Cavrois et al., 2017), cells undergoing viral fusion, as detected by the fluorescent CCF2 substrate in response to the release of the chimeric BlaM-Vpr protein, were sorted and compared in parallel to cells expressing the virally encoded, mass cytometry-compatible murine heat-stable antigen (HSA) marking productively infected cells. The comparison of these two independents single-cell datasets drew an atlas of CD4^+^ T cell phenotypic features contrasting entry and productive infection. Tonsillar T cells with features of memory, T_H_2, T_H_17 and T_REG_ subsets were thus found prone to viral entry in sharp contrast to naïve T cells whereas T_H_17 and T_FH_ were found predominant productively infected cells.

HIV-1 does not exclusively infect CD4^+^ T cells. Myeloid cells could also represent targets and/or facilitate viral dissemination although definitive *in vivo* confirmation of productive infection is still lacking. The best current technologies to detect HIV-1^+^ cells by flow cytometry call for a large number of cells difficult to obtain from blood, typically a poor source of mature myeloid cells. Microscopy studies using DNA/RNA FISH techniques are therefore better suited to address this detection challenge (Wang et al., 2012; Deleage et al., 2016; Estes et al., 2017). DNA/RNA FISH preserves tissue integrity and allows spatial interrogation of the microenvironment. This approach was used in a recent study (Estes et al., 2017) of multiple anatomic compartments in SIV and HIV-1 infection, further confirming that more than 98% of infected cells in primates would originate from lymphoid organs, a proportion likely similar in humans. A similar multiplex ISH microscopy method (mIFISH) has led to the identification of rare HIV-1^+^ CD21^+^ follicular dendritic cells (FDCs) and CD68^+^/CD163^+^ macrophages in lymph nodes of a viremic donor (Vasquez et al., 2018). Consistent with these results, confocal microscopy studies demonstrated that FDCs retain infectious HIV-1 in cycling endosomes through the complement receptor CD21 (Heesters et al., 2015) and multispectral flow cytometry (ImageStream) showed *in vitro* infection of macrophages via selective capture of HIV-1-infected CD4^+^ T cells (Baxter et al., 2014). While very powerful, microscopy imaging also has some drawbacks, including labor intensiveness, relatively low throughput, limited number of parameters on most instruments, and in most cases reliance on solid tissues that are hard to sample from human participants.

## HIV-1 Replication Cycle

The HIV-1 replication cycle has been studied for decades. Through the use of bulk methods, the processes governing viral replication were detailed to reach a canonical model (Engelman and Cherepanov, 2012). Single-cell technologies now reveal critical cell-to-cell disparities.

### Viral Entry

An innovative dually fluorescent viral platform combining lipophilic dyes staining viral membrane, and a cleavable GFP-Gag chimeric protein as a fluid-phase marker present inside the virion was developed to study HIV-1 entry (Miyauchi et al., 2009). This platform provided evidence that frequently occurring plasma membrane-fusion events were in fact dead-ends and suggested that only endosomal fusion is productive.

### Pre-integration Events

Co-detection of vRNA and vDNA by FISH-based technologies now enables detection of ongoing reverse transcription by high resolution microscopy, confirming its initiation in the cytoplasm (Puray-Chavez et al., 2017). Reverse transcription consistently culminated within a range of 10-14h post-entry in cell lines (Hulme et al., 2011; Holmes et al., 2015; Puray-Chavez et al., 2017). Several approaches enabled the visualization of the ill-characterized HIV-1 uncoating process: IN-TC/FlAsh (Arhel et al., 2006), A3F-YFP or IN-YFP (Burdick et al., 2017), or 5-ethynyl-2-deoxyuridine-labeled vDNA (EdU), GagiGFP, or CypA-DsRed/CA, with INsfGFP (Peng et al., 2014; Francis et al., 2016; Mamede et al., 2017; Francis and Melikyan, 2018a). Productive infection, attributed to only ≈2% of cell-bound-viruses (Burdick et al., 2017; Francis and Melikyan, 2018a), was characterized by tracking the intact or partially uncoated cores toward the nucleus where uncoating is completed in the vicinity of nuclear pores (Arhel et al., 2006; Francis et al., 2016; Mamede et al., 2017). While live-cell imaging of native viruses has not yet been achieved, snapshots of native virus egress in single primary cells are possible. Probing of the negative strand of the vDNA with a branching amplification technique along with protein staining enabled visualization of the nuclear import of native vDNA 12h post-infection confirmed the essential role of nuclear pore complex subunits (Chin et al., 2015).

### Integration

**FIGURE 4:**
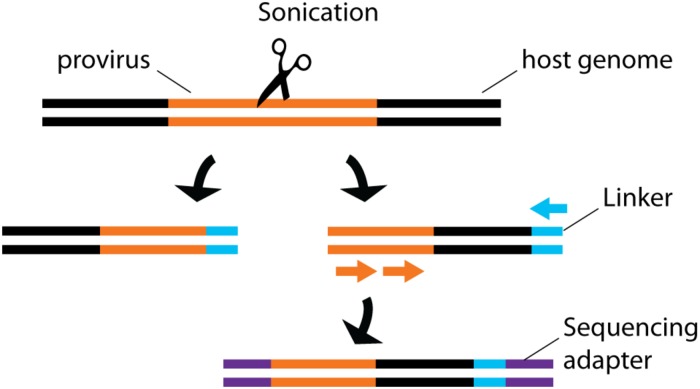
Schematic representation of integration site sequencing. Sonication produces random DNA cleavage sites across the host genome. Linker primers are ligated to provide a template for semi-nested provirus amplification. Sequencing primers are then ligated to allow sequencing. The random cleavage ensured by the sonication produced fragments of unique sizes, thus providing single-cell information.

By sequencing genomic vDNA from cell populations calculated to contain a single-infected cell, the frequency of HIV-1^+^ cells bearing a single integrated provirus was estimated at 85-90%, suggesting that only a minority of infected cells can sustain recombination, an important mechanism for viral evolution (Josefsson et al., 2011, 2013). To draw a landscape of HIV-1 integration sites in primary cells, the translocation-capture sequencing (TC-Seq) initially designed to study chromosomal rearrangements in B lymphocytes (Klein et al., 2011) was adapted in the integration sequencing assay (Cohn et al., 2015; Figure 4). Consistently with previous bulk population results (Schroder et al., 2002), this method detected globally more frequent integration events in intragenic regions of the genome with high transcriptional activity. Integration in genes with lower transcription activity occurred more frequently in treated individual with latent infection. While single-cell sequencing is a powerful way to map integrated vDNA in the genome, several imaging techniques were designed to assess its spatial location in the nucleus. They revealed that HIV-1 preferentially integrates in the chromatin found close to the nuclear membrane (Di Primio et al., 2013). Three-dimensional immuno-DNA FISH localized in *in vitro* infected primary CD4^+^ T cells both HIV-1 recurrent integration genes (RIGs) and integrated HIV-1 proviruses close to nuclear pores (Marini et al., 2015). More recently, a single-cell CRISPR imaging method was developed to assess integration in real-time. In this system, a guide DNA targeting the U3-LTR region triggers the co-localization of exogenous Cas9 proteins conjugated with two different quantum-dot fluorophores (Ma et al., 2017). This stringent dual-parametric detection provided an estimation of 1.6 ± 0.4 integrated events per HIV-1^+^ cell. This estimation was remarkably consistent with the data inferred by single-sorted cell sequencing results (Josefsson et al., 2011, 2013). Although initially applied to a cell line as a proof-of-concept, its compatibility with primary cell studies could provide physiological insight into vDNA integration.

### Assembly and Release

A key challenge in studying HIV-1 assembly and release is the inability to totally synchronize infection in target cells, underlining the need for single-cell technologies to conduct fine analyses. Total internal reflection fluorescence microscopy (TIRFM) enabled the visualization in adherent cell lines of clusters of mixed native and fluorescently tagged Gag proteins corresponding to single virions. Combined with photoconvertible fluorescence technology, it showed the nucleation of recently membrane-associated Gag proteins rather than the existence of long-lived stable platforms (Ivanchenko et al., 2009). Assembly was timed as fast as within 4-10 min after nucleation (Jouvenet et al., 2008; Ivanchenko et al., 2009), with an extra 25-min delay to achieve budding (Ivanchenko et al., 2009). The high resolution achieved by TIRFM or stochastic optical reconstruction microscopy (STORM) lead to the detection of ESCRT factors lattices co-localization with HIV-1 assembly sites (Baumgartel et al., 2011; Prescher et al., 2015). Other technologies enabling imaging of native virions on primary cells will be required to extend our knowledge of assemble/release in physiological context.

### Interplay With Host Cell Factors

More discovery-oriented approaches are required to understand the dynamics of the viral cycle in host cells *in vivo*, given the heterogeneity of immune cells. One strategy involves taking transcriptional snapshots of thousands of unsynchronized single cells and reconstitute the dynamic pattern of gene expression. This approach implies a compromise as the data produced only infer temporality to allow assessment of primary cells infected with native viruses. Using RT-qPCR on single-sorted cells, investigators were able to subdivide SIV replication cycle in various stages defined by the relative presence of multiply spliced versus unspliced vRNAs (Bolton et al., 2017; Tokarev et al., 2018). Simultaneous assessment of cellular gene expression revealed distinct transcriptional patterns associated with SIV infection, which could further be indexed to phenotypic data acquired during the single-cell flow cytometry sorting. This approach also provided some of the rare documented *ex vivo* evidence of SIV/HIV-1-mediated cellular protein downregulation. While in most cells progressive infection was associated with post-transcriptional downregulation of the CD4 protein, surface MHC class I expression was surprisingly largely maintained, in contradiction with previous reports suggesting maintenance of Nef-mediated MHC-I downregulation *in vivo* through selective pressure (Munch et al., 2001; Swigut et al., 2004). The same approach can in principle be further expanded to downstream unsupervised scRNA-Seq.

## Regulation of HIV-1 Expression

High resolution information is now available on the temporal regulation of viral gene expression. Live cell imaging studies of viruses encoding a fluorescent marker in place of the early gene *nef* or in frame within the late gene *gag* determined that as much as 42h can be necessary to complete a single replication cycle. This timespan varies considerably from one cell to another, suggesting the existence of cellular specificities that may temporally modulate viral egress (Holmes et al., 2015). Multiple well-tuned delays were noticed, including a 3-h delay between the onset of early and late gene translation followed by an overshadowing 6-12-h delay until viral assembly and release. These “programmed” delays would allow viruses to carry out pre-requisite processes that may be cell-specific. In independent studies, ddPCR provided *ex vivo* evidence of multiple blocks through transcription preventing initiation, elongation and termination of viral transcripts in CD4^+^ T cells (Yukl et al., 2018). Accumulation of multiply spliced variants sustaining only expression of the early Tat, Rev or Nef genes was also reported in some cells (Yukl et al., 2018). However, whether the transcriptional blocks and the well-tuned delays are related processes remain unclear because ddPCR does not preserve single-cell information and natively infected primary CD4^+^ T cells cannot be adequately interrogated with the currently available imaging methods. Indeed, single-cell PCR methods have the best potential to test this relation in physiological context.

A fine balance in the expression of the HIV-1 transactivator Tat protein is necessary to sustain adequate viral gene expression. As Tat is self-regulated, its downmodulation creates a transcriptional contraction that can lead to latency, a reversible state characterized by low or absent viral transcription. The dynamics of HIV-1 latency is complex and calls for high-dimensional tools (Kok et al., 2017). A time-lapse single-molecule mRNA fluorescence *in situ* hybridization (smFISH) method brought molecular evidence that Tat can act as a molecular switch. By following the relative expression of dual reporters of spliced and unspliced vRNA, a novel post-transcriptional mechanism of noise suppression stabilizing the commitment of HIV-1 to its active gene expression state was discovered (Hansen et al., 2018). The accumulation of unspliced transcripts reduced the relative level of Tat and Rev-coding spliced transcripts, creating a negative-feedback loop of noise suppression. Transcriptional noise would hinder fate commitment to the active state, thus promoting latency. A similar pattern of spliced/unspliced transcript temporal dynamics was observed in another single-cell study using a dual cherry/GFP reporter construct (Holmes et al., 2015).

While not all infected CD4^+^ T cells have the potential to become latent reservoirs, defining the determinants of this transition has been challenging in spite of intense investigation. The paucity of latent reservoirs persisting on suppressive ART *in vivo* and their difficult identification are major hurdles in the field. A number of latency models were used to circumvent this limitation (reviewed in Whitney and Brad Jones, 2018). While such models have enabled significant progress, they still face major questions regarding their representativity of actual events occurring *in vivo* and present notable discrepancies (Spina et al., 2013). The location of provirus integration was suggested as a key determinant for establishment of latency (Jordan et al., 2001; Sherrill-Mix et al., 2013). In that regards, barcoded HIV-1 Ensembles (B-HIVE) proved very informative. This technology involves insertion of a barcode into the viral genome. Because multiple viruses sharing the same degenerate 20 nucleotides-long barcode are extremely improbable, each barcode is statistically indicative of a single provirus providing single-cell resolution. After sequencing, vRNA can be connected to integrated vDNA sharing the same unique barcode, thus giving a robust examination of the transcriptional potential of each integration site (Chen et al., 2017, 2018). Using B-HIVE, latent proviruses were found integrated far from active host promoters or enhancers (Chen et al., 2017). This is consistent with integration sequencing data demonstrating that latent cells are more likely to bear vDNA in intergenic regions or in genes with low or no level of transcriptional activity than active reservoir cells. Proviruses inserted into active regions of the genome were found selected against probably due to virus-mediated toxicity, thus precluding the expansion of the clones bearing vRNA with the strongest transcriptional potential (Cohn et al., 2015).

Why would an unfavorable site of integration forcing latency would suddenly become good enough to fuel viral rebound? The regulation of viral gene expression by the intracellular environment can explain this apparent paradox. This notion is supported by the finding that timely infection of activated cells in the process of becoming quiescent promotes latency (Shan et al., 2017). Two recent scRNA-Seq studies using post-activation latency models drew transcriptional landscapes of quiescent HIV-1 infected cells (Bradley et al., 2018; Golumbeanu et al., 2018). Globally, latently infected cells clustered close to uninfected cells, suggesting that latent infection does not extensively remodel host cells (Bradley et al., 2018). Consistent with integration sequencing data, poor HIV-1 gene transcription was also associated with increase proliferative capabilities and cell survival (Bradley et al., 2018). A superficial resting state prone to HIV-1 reactivation could be discriminated from a deeper hardly reactivable latency by their higher expression level of genes associated with metabolism, gene expression, disease, immune system and DNA repair, giving rise to a 134-gene-specific transcriptional signature of inducible latent cells (Golumbeanu et al., 2018). Although powerful and informative with regard to the gene profile and mechanisms behind latency, these studies still rely on latency models with engineered laboratory-adapted viral strains which may not express all viral genes adequately, thus having an impact on the overall transcriptional landscape. As such, the extent at which the aforementioned findings can be transposed to *in vivo* latency is still unclear. Nevertheless, combined multi-faceted single-cell studies can lead to an elegant model of latency. Integration in intergenic regions far from enhancers or promoters results in a dead-end deep latency state. Inversely, integration in intragenic regions close to active enhancers or promoters leads to robust gene expression and selection against by cell-mediated cytotoxicity or virus-mediated apoptosis. Proviruses integrating in the perfect sweet-spot between those two extremes can become reversibly latent and undergo homeostatic expansion once the global transcriptional activity of its host decreases toward quiescence. It is still unclear if the distance of the integrated provirus from the nuclear membrane may also influence latency. The single-cell imaging tools to address this question are there however, and further investigations will certainly be informative in this regard.

Latency is not a permanent state. This is a central problem in HIV-1 pathogenesis and clinical care as the virus almost always spontaneously rebounds after treatment interruption. However, this problem can also become an opportunity: upon reactivation of latent reservoirs, the immune system can much better detect infected cells and destroy them. Therapeutically inducing reservoir reactivation to facilitate its elimination, also termed a “shock and kill” approach, is thus considered as potential strategy for HIV-1 cure. TCR cross-linking and PMA/ionomycin are well known to reactivate latent reservoirs *in vitro*, but their pleiotropic effects prohibit their use *in vivo*. A growing list of pharmaceutical compounds are now known as latency reversal agents (LRA) (reviewed in Kim et al., 2018). A microfluidic single-cell-in droplet PCR (scdPCR) assay in which single cells are partitioned in lipid droplets for individualized PCR allowed enumeration of CD4^+^ T cells that produce unspliced (us)RNA and multiply spliced (ms)RNA upon LRA stimulation of primary CD4^+^ T cells from ART participants (Yucha et al., 2017). It revealed that reactivation induced by TCR cross-linking or the LRA Romidepsin is asymmetrical at the single-cell level and is variable amongst donors. These results highlighted a fact that bulk analysis could not identify: latency reversal can be the result of a robust viral expression in a few cells or a modest induction in many (Yucha et al., 2017). The B-HIVE assay further shed light on latency reversal: cells responding to Vorinostat harbored integrated proviruses closer to enhancers than PHA did (Chen et al., 2017). The insertion context defined at the single-cell level thus carries some predictive value about the potential response of a provirus to LRAs.

Flow cytometric RNA FISH assays, thanks to their ability to simultaneously monitor at the single-cell level vRNA and HIV-1 protein expression upon LRA reactivation, are powerful approaches in latency reversal studies (Baxter et al., 2016, 2018; Grau-Exposito et al., 2017, 2019). For example, while Romidepsin increased frequencies of vRNA^+^ cells, this LRA was a poor inducer of Gag protein expression in these reactivated cells compared to PMA/ionomycin (Grau-Exposito et al., 2017). The kinetics of latency reversal at the transcriptional and translational level could also be monitored using this method (Martrus et al., 2016). As detection of HIV-1^+^ cells can be combined with multiparametric phenotyping for cellular markers, RNAflow-FISH approaches can distinguish subsets of CD4^+^ T cells able to respond to LRAs in primary clinical samples. For example, the protein kinase C (PKC) agonist Bryostatin-1 preferentially reactivated T_EM_ reservoirs whereas the PEP005 showed broader activity, including on T_CM_ cells (Baxter et al., 2016) and stem-cell memory T cells were found to be more refractory to reactivation (Grau-Exposito et al., 2019). These are early studies for these technologies that suggest they have potential as advanced monitoring tools for clinical trials.

## Viral Reservoirs

Determining the size of HIV-1 reservoir during ART is challenging, as long-lived latently infected cells are largely transcriptionally silent. Early methods applied to bulk populations relied on the direct detection of total or integrated HIV DNA. Few DNA-based detection methods can accurately distinguish the rare cells bearing an intact, potentially replicative-competent provirus from the vastly more numerous HIV-1^+^ cells bearing integrated proviruses containing lethal defects. Intact proviruses were found enriched in cell refractory to standard *in vitro* stimulation with frequencies in the few percent range of total vDNA (Ho et al., 2013). While the potential of these viruses to reactivate *in vivo* is unknown, they may constitute a higher barrier to cure. Conversely, the quantitative viral outgrowth assay (Q-VOA), in which single infected-cell are seeded in limiting dilutions among reporter cells to allow the amplification of p24, gives a minimal estimation of the size of replication-competent reservoirs. These methods offer the highest and lowest estimates of “total” and “replication-competent” viral reservoirs. New highly sensitive and specific flow cytometry-based methods based on detection of viral products (viral RNA and/or proteins) provide additional information on the competence of the reservoirs at the single-cell resolution required to associate viral or cellular features to the quantified reservoirs, with estimated VR sizes that are intermediate between standard DNA and Q-VOA quantification (DeMaster et al., 2015; Baxter et al., 2016, 2018; Grau-Exposito et al., 2017; Zhang et al., 2018; Pardons et al., 2019). In these studies, translation-competent reservoirs were determined by the expression of p24 (Baxter et al., 2016, 2017a; Pardons et al., 2019) whereas production of viral RNAs such as *gagpol* defined transcription-competent reservoirs (Baxter et al., 2016; Grau-Exposito et al., 2017). To isolate live reservoir cells, dual staining with broadly neutralizing antibodies (bNAbs) has also been successfully applied (Cohn et al., 2018). These methods are well adapted to assess HIV-1^+^ events in blood, where reservoir cells are rare but cell numbers is not limiting. Probing lymphoid tissues, the primary viral sanctuaries during ART, is more difficult because of limited sample availability. While cytometry can be performed on extracted cells, *in situ* microscopy is frequently the method of choice to perform those measurements in tissues. Signal amplification technologies have been developed to allow simultaneous single-cell detection of proviral vDNA and vRNA, giving valuable information both on DNA integration and viral gene expression without altering tissue structure (Deleage et al., 2018). Coupled with automated imaging of multiple tissues, these approaches have allowed rigorous assessment of anatomical compartmentalization and total body burden of SIV and HIV-1 reservoirs (Deleage et al., 2016; Estes et al., 2017, 2018). A variant of this method enabled detection of spliced viral RNA using probes specific for the *tat-rev* splice junctions, thus increasing the likelihood of detecting viral RNA^+^ cells *in situ* associated with replication-competent viruses (Deleage et al., 2018). Applied to flow cytometry, this approach distinguished spliced and unspliced vRNA and was sufficiently sensitive to capture the delayed export from the nucleus to the cytoplasm of unspliced vRNA compared to the spliced variant (Puray-Chavez et al., 2017). Determining if residual vRNA^+^ cells can represent a primary source of viral rebound upon treatment interruption necessitates single-cell analysis because expanded clones cannot be recognized as such in bulk analyses. The cell-associated HIV-1 RNA and DNA, single-genome sequence assay (CARD-SGS) can connect residual HIV-1 transcriptional activity to proviruses in ART-treated donors (Wiegand et al., 2017). Single-cell resolution of sequencing is achieved through statistical assumption of the dilution required to obtain single HIV-1^+^ cells. On ART, vRNA sequences were found less diverse than vDNA, further suggesting that few HIV-1^+^ cells actually contribute to the viral diversity *in vivo* (Wiegand et al., 2017). The extent of the contribution of expanded vRNA^+^ clones to viral rebound is still debated, as there are notable discrepancies between reported observations (Cohn et al., 2015; Barton et al., 2016; Boritz et al., 2016; Kearney et al., 2016). Nevertheless, all these studies are consistent with a very limited pool of HIV-1^+^ cells fueling viremia or viral rebound. These findings emphasize the importance of studying proviral sequences at the single-cell level to discriminate the cells susceptible to fuel viremia from the numerically superior ones that cannot.

## Immune Response

HIV-1 pathogenesis is determined by complex interactions between the virus and the host immune system. While the study of the anti-HIV-1 immune response quickly became a major field of research in the perspective of developing effective cure strategies and prophylactic vaccines, it is only relatively recently that single-cell technologies have been exploited and have yielded major results in this area.

### Immune Response and Immune Dysfunction

Multiple arms of the immune responses are dysregulated in HIV-1 infection. Hypergammaglobulinemia is frequent, contrasting with qualitative defects of humoral immunity such as lower titers and less durable B cell responses to seasonal influenza vaccine (Tebas et al., 2010; Crum-Cianflone et al., 2011; de Armas et al., 2019). The extreme diversity of the B cell receptor (BCR) repertoire and complex differentiation patterns of B cell subsets limit the insight gained from bulk population studies. To overcome these limitations, transcriptional profiling was recently performed by scRNA-Seq on post-vaccination Influenza-specific memory B cell in virally suppressed HIV-infected individuals (de Armas et al., 2019). In this approach, fluorescent probes identifying HA-specific B cells allowed single-cell sorting and downstream scRNA-Seq. The high-dimensional data thus generated contrasted transcriptional differences in cells otherwise indistinguishable by conventional flow cytometry. *PTEN*, a gene associated with hampered BCR signaling through inhibition of the PI3K signaling pathway, was found elevated in influenza-specific B cells from HIV-1-infected individuals. Other studies attributed B cell dysfunction to inefficient help provided from germinal center follicular T cells (GC T_FH_) (Cubas et al., 2013, 2015; Boswell et al., 2014). The analysis of lymph node samples from untreated HIV-1^+^ donors by combined high dimensional mass cytometry and TCR repertoire single-cell sequencing shed light on the fine structure of these T_FH_ responses (Wendel et al., 2018), revealing that HIV-1-specific T_FH_ expand but become functionally skewed with limited TCR diversity, features that correlate with B cell dysregulation in the same lymph node.

T cell dysfunction is a hallmark of chronic infections, and is in part an adaptive compromise required for the host by antigen persistence, as it balances some partially effective immunity with reduction of immunopathology. While this immune impairment was initially conceptualized as a state of chronic loss of function, a number of studies have highlighted T cell “exhaustion” as being a distinct differentiation program, which itself presents important cellular heterogeneity among subsets of exhausted cells (e.g., CD4^+^ vs CD8^+^ T cells, McLane et al., 2019). High dimensional single-cell analyses can now provide a better understanding of this complexity. Rather than relying on a limited set of parameters to identify exhausted T cells in HIV-1 infected humans, investigators used an epigenomic-guided mass cytometry approach (Bengsch et al., 2018b) and identified up to 12 exhausted CD8^+^ T cell clusters with considerable heterogeneity in inhibitory co-receptor and transcription factor co-expression. Some clusters of severely exhausted CD8^+^ T cells were found similarly enriched in people afflicted with lung cancer whereas others were differentially represented, suggesting that a common core biology of T cell exhaustion across diseases exists along with more disease-specific defects (Bengsch et al., 2018b). The extent to which these findings also apply to CD4^+^ T cells remains to be determined, and single-cell technologies applied to CD4^+^ T cell biology will be informative. Mass cytometry data revealed a complex network of CD4^+^ T cells clusters that correlated with functional decline and was associated with late ART initiation (Bekele et al., 2019). Most high-dimensional studies on T cell dysfunction in HIV infection have thus far been focused on T cell subsets, not HIV-specific T cells, a step required to delineate antigen-specific immune dysfunction from broader dysregulation in the context of HIV-1 infection and associated chronic immune activation.

While epitope-specific tetramers of good quality and broad HLA Class I diversity are easily accessible for human CD8^+^ T cell studies and can also be used for high-dimensional flow cytometry or mass cytometry studies (Newell et al., 2013), accessibility to reliable human Class II multimers remains limited. Several groups have thus established sensitive methods based on upregulation of activation-induced markers (AIM) to detect virus-specific CD4^+^ T cells after cognate antigen stimulation (Zaunders et al., 2009; Havenar-Daughton et al., 2016; Reiss et al., 2017). A major advantage of this approach is the possibility to live-sort HIV-1-specific CD4^+^ T cells for downstream analyses such as -omics studies, including single-cell technologies. This was recently illustrated by a genome-wide transcriptome profiling study of HIV-1-specific CD4^+^ T cell responses pre- and post-ART (Morou et al., 2019). Expression patterns of selected genes and their association with cell phenotypes was confirmed at the single-cell level by multiplexed RNAflow-FISH, providing an experimental pipeline for detailed HIV-1-specific CD4^+^ T cell studies. Compared to Thelper responses identified in HIV-1 elite controllers, ART did not fully reverse the dysregulated transcriptional program identified in viremic progressors before initiation of therapy (Morou et al., 2019). This is consistent with mass cytometry studies of the total CD8^+^ and CD4^+^ T cell subsets conducted on other HIV-1^+^ cohorts, which showed incomplete restoration of clusters of exhausted T cells on suppressive ART (Bengsch et al., 2018b; Bekele et al., 2019). A precise map of the corrected versus persistently altered gene modules will be key to understand residual T cell dysfunction in people living with HIV-1.

Single-cell technologies can also facilitate in-depth studies of anatomic compartments for which sampling is quite limiting in humans. Until recently, the paradigms of protection against HIV-1 largely relied on peripheral blood studies, although virus replication occurs mainly in lymphoid tissues. An approach combining high dimensional mass cytometry, scRNA-Seq, and ATAC-Seq enabled transcriptional and epigenetic-profiling of a novel extrafollicular LN-resident CD69^+^ virus-specific CD8^+^ T cell subset (Buggert et al., 2018), with notable transcriptional and functional differences observed compared to blood HIV-1-specific CD8^+^ T cells. Central nervous system (CNS) studies exemplify the challenges of human studies as well as the potential to make optimal use of the rare cell populations isolated from precious clinical samples. CNS involvement remains a significant issue, as neurocognitive disorders occur in spite of highly effective ART and as the CNS can serve as immune sanctuary. A recent study used scRNA-Seq to define the immune cell landscape in the cerebrospinal fluid (CSF) of virologically suppressed individuals (Farhadian et al., 2018). They found a rare subset of myeloid cells whose gene signature overlapped with neurodegenerative disease-associated microglia. These findings suggest that an immunopathogenic subset of myeloid cells may perpetuate neuronal insults during HIV-1 infection, thus providing physiological evidence of myeloid dysfunction. Unsupervised analytical approaches have the benefits of identifying new subsets of rare dysregulated cells, better assessing immune cell dysfunction *in vivo* and identifying factors with direct contribution to residual immune impairment. Clearly, further investigations will be needed to establish the extent of myeloid dysfunction during HIV-1 infection.

### Vaccine Development and Immunomonitoring

The best hope to control the HIV-1 pandemic probably resides in prophylactic vaccines. Although none of the attempted vaccine trials led to a definitive breakthrough, correlates of protection could be identified in human studies (reviewed in Corey et al., 2015). Broadly neutralizing Abs (bNAbs) targeting Env epitopes from many HIV-1 strains exist in a small proportion of chronically infected individuals (reviewed in Klein et al., 2013; Kwong et al., 2013; Burton and Mascola, 2015). However, how to elicit bNAb-producing B cells by vaccination strategies remains unclear because the ontogeny of this atypical B cell response is not yet fully elucidated, and may be very challenging to elicit by a vaccination strategy. Indeed, bNAbs originate from rare clones diluted in the vastly heterogeneous B cell populations, which precludes the use of bulk analytic approaches. The B cell repertoire was studied by combining image-based on–chip cytometry and micro engravement (Ogunniyi et al., 2014; Figure 4). Thousands of independent cells loaded into microwells were labeled with antibodies, then subjected to RT-PCR and sequencing, thus yielding single-cell phenotypic information and sequencing data in the same system. RT-PCR on single-sorted B cells emerged as an essential tool to understand the features of bNAb generation (Scheid et al., 2009; Sundling et al., 2012, 2014; Wang et al., 2016). In contrast to bulk population PCR, this approach readily identified unique Env-specific B cell clones and provided the matched heavy and light V(D)J sequences for subsequent cloning, thus allowing the functional characterization of key monoclonal antibodies *in vitro* (Sundling et al., 2012, 2014). This approach is used to conduct preclinical assessment of vaccine candidates in non-human primates (NHPs) (Scheid et al., 2009; Sundling et al., 2012, 2014; Wang et al., 2016), and to explore the pathways toward bNAb development in humanized mouse models upon sequential immunization (Escolano et al., 2016). Furthermore, single-cell BCR sequencing of naïve B cells in HIV-uninfected human donors has been successfully used to estimate the frequencies of germline precursors that would have the potential to develop into a given bNAb lineage, provided that an optimal vaccination strategy could lead them along this path (Jardine et al., 2016). Deployment of such advanced single-cell technologies in Phase I clinical trials of new immunogens should help select the most promising vaccination strategies for further development.

Single-cell transcriptomics and epigenomics strategies, in some cases combined with TCR sequencing, have also been successfully applied to study the T cell response in human diseases (Buggert et al., 2018; Jerby-Arnon et al., 2018). Although the conduct of such single CD4^+^ or CD8^+^ T cell studies currently appear to lag behind in the HIV field compared to B cell studies and to investigations of cellular immunity in other diseases, the conceptual and technical frameworks now appear to be mature for such cutting-edge investigations in advanced HIV-1 vaccine immunomonitoring.

Standard biostatistical approaches often failed to appreciate differences in the quality of the immune vaccine response because they rely on expected biological outcome and consequently fail to grasp the inherent complexity of the multi-component nature of immunity. Single-cell technologies have been used to provide high throughput multi-dimensional data, but the lack of computational tools has in many instances led to suboptimal exploitation of the wealth of data generated. To overcome these limitations, analytical frameworks harnessing the full extent of single-cell technologies were tested on multidimensional datasets (Finak et al., 2014; Lin et al., 2015). For example, combinatorial polyfunctionality analysis of antigen-specific T cell subsets (COMPASS) models enable identification of cell subsets and select those most likely to have antigen-specific responses using a Bayesian hierarchical framework, thus allowing to correlate the quality of an individual response with clinical outcome. COMPASS identified CD4^+^ T cell polyfunctionality as a new correlate of vaccine efficacy in the RV144 HIV vaccine trial and delineated qualitative differences in CD4^+^ T cell responses between different HIV-1 vaccine regimens (Lin et al., 2015). These findings support the hypothesis that the general quality of response is more important to determine the outcome of vaccination, and perhaps infection, than magnitude on single-parameter responses (Lin et al., 2015). Such studies further support combining single-cell technologies with multivariate computational analyses to adequately interpret the complex immune network at play during infection or vaccination.

## Perspectives

Single-cell analysis is not a novel concept. However, recently developed technologies are now bringing the resolution and depth of single-cell investigations to the next level in every field of biology. Immunology and cancer are fields that pioneered the use of these new tools. HIV-1 research contributed to the development of many technologies in the past, for example single-cell microscopy applied to investigation of the viral replication cycle. Yet, compared to other areas of biomedical research, the field appears to currently lags behind in fully adopting newer high throughput single-cell technologies, this despite the fact that the very nature of HIV-1 biology would extensively benefit from these tools. The ball is now in the HIV-1 researchers’ court.

## Author Contributions

GS and MD performed the literature review and wrote the manuscript. DK edited the manuscript and provided supervision. All authors read and approved the final version of the manuscript.

## Conflict of Interest

The authors declare that the research was conducted in the absence of any commercial or financial relationships that could be construed as a potential conflict of interest.
